# Supplementary material for: Trends in cardiac rehabilitation rates among patients admitted for acute heart failure in Japan, 2009–2020
Source: PLoS One. 2023 Nov 28;18(11):e0294844. doi: 10.1371/journal.pone.0294844 (PMC10684100; doi:10.1371/journal.pone.0294844)

**Supplemental Material**

Fig A. Inpatient CR rates by age from 2009-2020


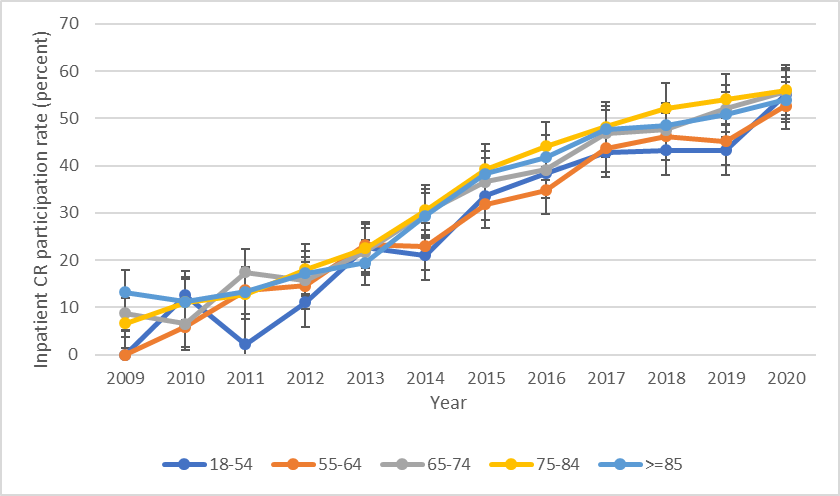


Fig B. Inpatient CR rates by NYHA class from 2009-2020


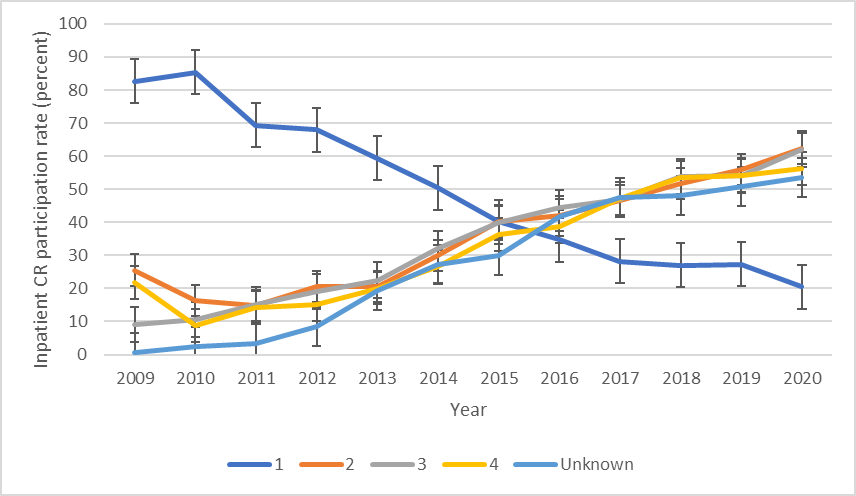


Fig C. Inpatient CR rates by BMI from 2009-2020


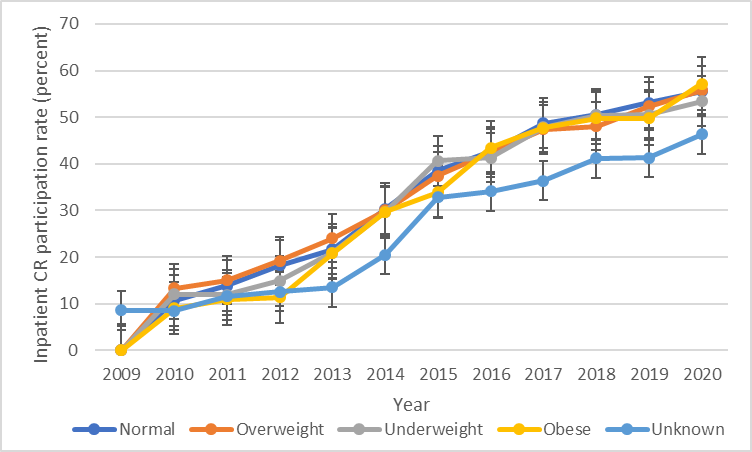


Fig D. Inpatient CR rates by smoking status from 2009-2020


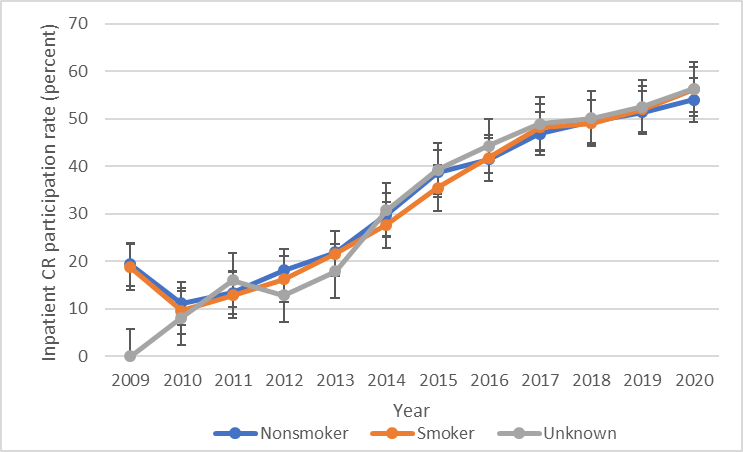


Fig E. Inpatient CR rates by diabetes status from 2009-2020


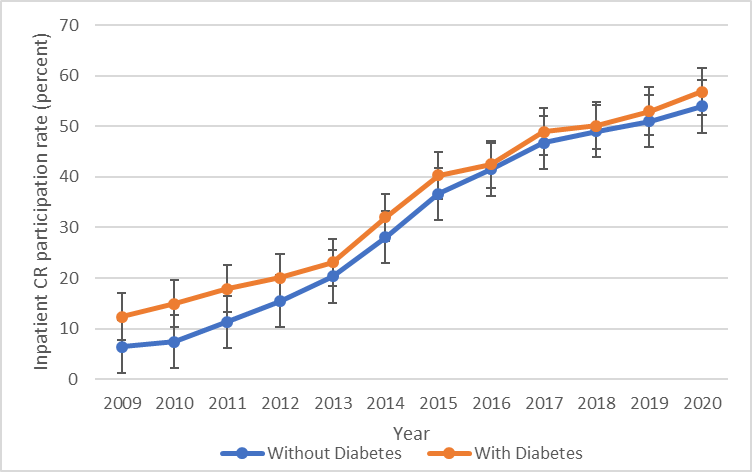


Fig F. Inpatient CR rates by AF status from 2009-2020


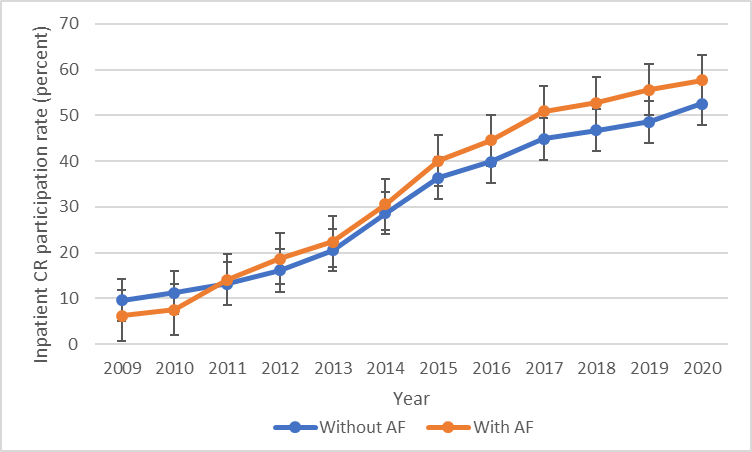

Supplement: S1 File — (DOCX) [file pone.0294844.s001.docx]
